# Assessment of right ventricular function by treadmill exercise stress cardiovascular magnetic resonance in patients with repaired tetralogy of Fallot

**DOI:** 10.1186/1532-429X-16-S1-O105

**Published:** 2014-01-16

**Authors:** Juliana Serafim da Silveira, Leah Geyer, Jason Craft, Subha V Raman, Orlando P Simonetti, Stephen C Cook

**Affiliations:** 1 The Ohio State University, Columbus, Ohio, USA; 2 University of Cincinnati, Cincinnati, Ohio, USA; 3 University of Pittsburgh, Pittsburgh, Pennsylvania, USA

## Background

Cardiac MR (CMR) is considered the gold standard to assess right ventricular (RV) function in different scenarios, including cases of congenital heart disease. Moreover, an MR-compatible treadmill coupled with Real Time (RT) CMR has been developed for stress testing, and may be an optimal choice in this setting. The aims of this study were to determine the feasibility and accuracy of a RT CMR protocol to evaluate RV function at rest and after treadmill exercise in adults with history of repaired Tetralogy of Fallot (ToF).

## Methods

Ten young adults (median age 29 years, range 19-47) were prospectively enrolled, and imaging was performed using a 1.5T scanner (Avanto, Siemens) and 32-channel phased array coil. RV cavity was covered by 10 to 12 contiguous 8 mm transverse slices. ECG-Gated SSFP cine was performed at rest (TE 1.3 ms, TR 2.7 ms, temporal resolution 48 ms, α = 75°, BW 930 Hz/pixel), while non-triggered RT cine was executed both at rest and after stress (TE 1.0 msec, TR 2.3 msec, temporal resolution 46 ms, α = 65°, BW 1488 Hz/pixel). Patients underwent treadmill exercise with 12-lead ECG (Cardiosoft, GE) to achieve a goal of ≥ 85% age-predicted maximum heart rate (APMHR). RT cine imaging was performed immediately upon cessation of exercise. End-diastolic (EDV) and end-systolic (ESV) volumes were calculated by two different observers using Simpson's rule by manually tracing RV endocardial borders. Interobserver agreement was analyzed by the Pearson's correlation coefficient whereas differences were assessed with paired t-test (SPSS 22.0, IBM).

## Results

**Figure 1:**
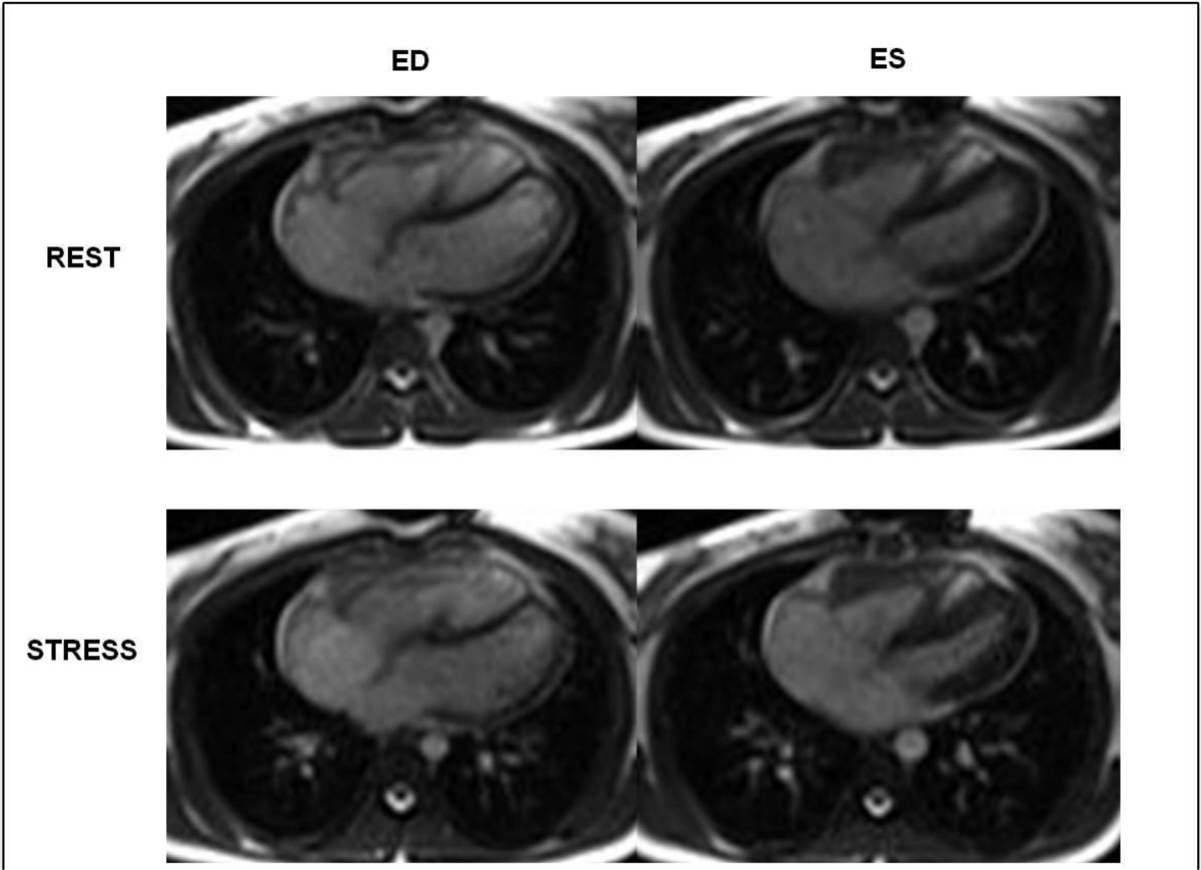
**End-diastolic (ED) and end-systolic (ES) Real Time cine images acquired in the transverse plane from a patient who demonstrated appropriate contractile reserve in response to exercise**. There is a decrease in right ventricular ES volume during stress compared to rest, resulting in an increase in ejection fraction. Note that there is no significant change in right ventricular ED volume from rest to stress.

Both ECG-Gated and RT techniques demonstrated excellent interobserver agreements for EDV, ESV, and ejection fraction (EF) (Correlation coefficients of 0.99 in all scenarios, p < 0.01), indicating good reproducibility. Average results between the two observers are listed in Table 1. At rest, there was no significant difference between ECG-Gated and RT EDV, ESV, and EF. The maximal heart rate after exercise was on average 189 ± 9.5 bpm. Seven patients reached at least 85% of APMHR to a maximal heart rate of 97.4% ± 3.3%. ECG data revealed no arrhythmias or ST segment changes during exercise. In 6 patients the RVEF significantly increased (Δ10.2 ± 6.8%, p-value 0.014) which was mainly attributed to a decrease in RV ESV (Δ38.5 ± 26.1 ml, p-value 0.015), suggesting good RV contractile reserve in these patients (Figure 1). In 4 patients, the RVEF did not increase (Δ2.7 ± 2.1%, p-value 0.086). Neither the RV ESV nor EDV appreciably changed (Δ0.3 ± 0.7 ml, p-value 0.486; and Δ12.1 ± 10.3 ml p-value 0.100, respectively). These patients were considered to have poor contractile reserve.

**Table 1:** Right ventricular mean volumes and function obtained at rest and after stress, measured by ECG-Gated and Real-time sequences.

	Rest			Stress
	ECG-Gated	Real-time	P-value	Real-time
EDV (mL)	250.5 ± 72.2	253.8 ± 81.3	0.63	226.3 ± 85.2
ESV (mL)	131.8 ± 45.6	132.6 ± 52.8	0.82	109.4 ± 56.3
EF (%)	48.1 ± 6.2	48.9 ± 6.0	0.48	54.0 ± 10.2

Abbreviations: EDV, end diastolic volume; ESV, end systolic volume; EF, ejection fraction.

## Conclusions

The feasibility of RT CMR imaging coupled with treadmill exercise was demonstrated. The diagnostic value of this technique to assess RV function in patients with repaired ToF deserves further assessment in a larger patient cohort.

## Funding

Partially funded by NIH: R01 HL102450.

